# Age-related exacerbation of lung damage after trauma is associated with increased expression of inflammasome components

**DOI:** 10.3389/fimmu.2023.1253637

**Published:** 2024-01-11

**Authors:** Yuzhuo Zhou, Fanshuai Meng, Kernt Köhler, Jasmin Maria Bülow, Alessa Wagner, Claudia Neunaber, Katrin Bundkirchen, Borna Relja

**Affiliations:** ^1^ University Ulm, Department of Trauma, Hand, Plastic and Reconstructive Surgery, Translational and Experimental Trauma Research, Ulm, Germany; ^2^ Hannover Medical School, Department of Trauma Surgery, Hannover, Germany; ^3^ Uniklinik RWTH Aachen, Department of Trauma and Reconstructive Surgery, Aachen, Germany; ^4^ Justus Liebig University Giessen, Institute of Veterinary Pathology, Giessen, Germany

**Keywords:** femur fracture, hemorrhagic shock, aging, pulmonary injury, inflammasome, NF-KappaB

## Abstract

**Background:**

Trauma, a significant global cause of mortality and disability, often leads to fractures and hemorrhagic shock, initiating an exaggerated inflammatory response, which harms distant organs, particularly the lungs. Elderly individuals are more vulnerable to immune dysregulation post-trauma, leading to heightened organ damage, infections, and poor health outcomes. This study investigates the role of NF-κB and inflammasomes in lung damage among aged mice post-trauma.

**Methods:**

Twelve male C57BL/6J mice underwent hemorrhagic shock and a femoral fracture (osteotomy) with external fixation (Fx) (trauma/hemorrhage, THFx), while another 12 underwent sham procedures. Mice from young (17-26 weeks) and aged (64-72 weeks) groups (n=6) were included. After 24h, lung injury was assessed by hematoxylin-eosin staining, prosurfactant protein C (SPC) levels, HMGB1, and *Muc5ac* qRT-PCR. Gene expression of *Nlrp3* and *Il-1β*, and protein levels of IL-6 and IL-1β in lung tissue and bronchoalveolar lavage fluid were determined. Levels of lung-infiltrating polymorphonuclear leukocytes (PMNL) and activated caspase-3 expression to assess apoptosis, as well as NLRP3, ASC, and Gasdermin D (GSDMD) to assess the expression of inflammasome components were analyzed via immunostaining. To investigate the role of NF-κB signaling, protein expression of phosphorylated and non-phosphorylated p50 were determined by western blot.

**Results:**

*Muc5ac*, and SPC as lung protective proteins, significantly declined in THFx versus sham. THFx-aged exhibited significantly lower SPC and higher HMGB1 levels versus THFx-young. THFx significantly increased activated caspase-3 versus both sham groups, and THFx-aged had significantly more caspase-3 positive cells versus THFx-young. IL-6 significantly increased in both sham and THFx-aged groups versus corresponding young groups. THFx significantly enhanced PMNL in both groups versus corresponding sham groups. This increase was further heightened in THFx-aged versus THFx-young. Expression of p50 and phosphorylated p50 increased in all aged groups, and THFx-induced p50 phosphorylation significantly increased in THFx-aged versus THFx-young. THFx increased the expression of inflammasome markers IL-1β, NLRP3, ASC and GSDMD versus sham, and aging further amplified these changes significantly.

**Conclusion:**

This study’s findings suggest that the aging process exacerbates the excessive inflammatory response and damage to the lung following trauma. The underlying mechanisms are associated with enhanced activation of NF-κB and increased expression of inflammasome components.

## 1 Introduction

Trauma is a major leading cause of death among young adults worldwide, whereby femur fracture being a significant type of traumatic musculoskeletal injury that increases mortality and can cause pulmonary complications such as acute respiratory distress syndrome (ARDS) (1, 2). Femoral fractures are often accompanied by hemorrhagic shock (HS) (3), that can reduce oxygen or nutrient supply to vital organs (4), leading to remote lung injury (RLI). Damaged tissues in a state of ischemia and hypoxia following HS can release damage-associated molecular patterns (DAMPs) and other inflammatory mediators like cytokines, including interleukin (IL)-1β or IL-6 (5–7). These biomolecules can activate and attract neutrophils, leading to apoptosis in the lungs (8–11). IL-6, a biomarker and key driver of injury-induced inflammation after trauma and HS (7, 12), can be transiently suppressed in a murine model by administering anti-IL-6 antibody, which resulted in reduced lung injury and suppression in the levels of key inflammatory mediators following trauma and HS (7). Trauma-induced pro-inflammatory changes can be concomitant with enhanced neutrophil infiltration and apoptosis, as well as general lung injury, and increased activation levels of the nuclear factor-kappa (NF-κ)B *in vivo* (12–14). Adjacent to the NF-κB signaling pathway, which comprises the p50/p65 and the inhibitor of κB (IκB) protein, it was shown that femoral fracture and hemorrhagic shock induced presence of pro-inflammatory markers, of which among others the inflammasome-activated IL-1β was associated with enhanced neutrophil infiltration into the lungs, apoptosis and general lung injury in mice (15). Trauma-induced oxidative stress and DAMPs can trigger the formation of the nucleotide-binding domain (NOD)-like receptor protein (NLRP)3 inflammasome (16, 17). Once initiated by stimuli, for instance endotoxin such as lipopolysaccharide, NLRP3 proteins polymerize in the NLRP3 inflammasome multiprotein complex, which in turn promotes the recruitment and activation of Pro-caspase-1, and thus, the maturation of IL-1β leading to inflammatory response (18). Reduced NLRP3 inflammasome-mediated IL-1β release and decreased caspase-3 concentrations, thus lead to inhibited apoptosis and pyroptosis alleviated pulmonary pathological damage and improved the survival rate of the sepsis mice *via* the MAPK/NF-κB/NLRP3 pathway (19).

The epidemiology of trauma-associated complications during the hospital stay continues to evolve. Recently, it was shown that an ageing population with increasing incidence of multiple organ failure, particularly in males, stay longer on intensive care units (ICU) and requires increasingly more hospital resources without improvement in survival (20). Aging is accompanied by an increase in circulating pro-inflammatory cytokines and pro-apoptotic factors, even in the absence of infection, suggesting a low-level chronic inflammatory state, known as “inflammaging” (21, 22). This state is evident in the elevated levels of circulating as well as pro-inflammatory cytokines in the lung, such as of IL-1β, IL-6, IL-8, and TNF-α (23, 24). The aging process leads to changes in primary and secondary lymphoid tissues, which causes the lung tissue to become less flexible, and decreasing the lung’s ability to respond to trauma (23). Moreover, there is a higher activity of caspase-3 in aged individuals’ heart, which further exacerbates the problem (25). Unbiased genome-wide analyses exposed age/stress-related epigenetic effects with a proinflammatory profile and altered NF-κB-related gene networks (26).

Thus, the pulmonary environment becomes more inflammatory with increasing age (27, 28). Interestingly, there is an increased NLRP3 inflammasome activity in aged and stressed lungs, which increased the expression of mature IL-1β in aged individuals (29, 30). These age-related changes in innate and adaptive immunity suggest that aged individuals may not respond to immunological threats such as trauma as effectively as younger individuals. However, the exact mechanistic drivers of aging-related inflammatory modulations in trauma are not entirely understood. Based on this background, the study aimed to investigate the effect of aging on inflammatory changes and the underlying mechanisms that cause lung injury in mice after femur fracture and hemorrhagic shock, with a distinct focus on the NF-κB signaling pathway as well as on the expression of inflammasome components.

## 2 Materials and methods

### 2.1 Animal husbandry

All animal studies were carried out in strict accordance with the German Animal Law and with the permission of the local authorities in Lower Saxony, Germany (approval number: 33.12-42502-04-17/2491). Young (17 - 26 weeks) and aged (64-72 weeks) male C57BL/6J mice (Janvier Labs, Le Genest-Saint-Isle, France) were used for the experiments (31). The animals were kept in individual cages under standardized conditions at the Central Animal Laboratory of the Hannover Medical School, where cages, bedding and drinking bottles were frequently changed and standard softwood granules (Altromin GmbH, Lage, Germany) for experimental animals were used as litter material.

### 2.2 Group distribution

24 male C57BL/6J mice held in conventional animal room were randomly assigned to one of the four groups. Animals in sham groups (sham young: n = 6; sham aged: n = 6) received the femoral artery catheterization and an external fixator, but no osteotomy or blood loss were induced. In trauma groups (THFx young: n = 6, THFx aged: n = 6) hemorrhagic shock with resuscitation was induced and an external fixator followed by osteotomy (Fx) of the femur was applied (trauma/hemorrhage, THFx). Young mice were 17 - 26 weeks and aged mice were 64 - 72 weeks old. The animals were sacrificed 24 hours after experiment induction.

### 2.3 Experimental model

All surgical procedures were performed under deep inhalation anesthesia using isoflurane (Baxter Deutschland GmbH, Unterschleißheim, Germany) as described before (3, 31, 32). Surgical procedure was initiated after repeated periodic verification of the negative interphalangeal reflex in mice. Mice were kept warm during the procedure using a heating pad and the eyes were protected from drying out with Bepanthen eye ointment. An intraoperative analgesia with 5 mg/kg body weight carprofen (Zoetis inc.) and 1 mg/kg body weight butorphanol (Zoetis inc., USA) were injected subcutaneously (s.c.). Prilocaine hydrochloride Aspen Germany GmbH, Germany was used for local anesthesia at the surgical site. Metamizole (Ratiopharm GmbH, Germany) at 200 mg/kg bodyweight was mixed into drinking water for postoperative analgesia, and carprofen and butorphanol were injected s.c. according to indication. After surgery, the animals were placed under warmth and red light until full consciousness is regained, then housed in independent cages to avoid them behaving aggressively towards each other, which could affect the healing of the surgical wound. After surgery, animals were regularly controlled and assessed for vital signs and mobility. All surgical procedures were conducted as described before (3, 31, 32). In the sham and trauma groups, a catheter was inserted into the left femoral artery and an external fixator (MouseExFix simple L 100%, RISystem, Davos, Switzerland) was implanted into the right femur. Animals in the trauma groups (THFx young; THFx aged) underwent a pressure-controlled hemorrhagic shock (HS). In brief, after insertion of the catheter into the left femoral artery, blood was collected until the mean arterial blood pressure reached 35 ± 5 mm Hg. The hypovolemic state of shock was maintained for a total of 90 minutes. The animals were then re-infused with four times the amount of blood drawn (up to maximum 2.4 ml) using body warm Ringer’s solution within 30 minutes and afterwards the catheter was removed. The external fixator was placed, and diaphysis was osteotomized in THFx groups centrally between the two middle pins using a 0.44 mm diameter wire saw (Gigly wire saw, RISystem). Prolene 6-0 (Ethicon, Cincinnati, USA) was used to suture the wounds and the animals were allowed to move freely immediately after the experiments were completed.

### 2.4 Harvesting procedures

After 24 hours of surgery, the animals were euthanized with an intraperitoneal injection of 75 mg/kg body weight of ketamine (Zoetis inc.) and 1 mg/kg body weight of medetomidine (Zoetis inc.). The abdominal cavity was opened, and a heparinized sharp 25-gauge syringe was used to puncture the heart for blood collection, followed by cervical dislocation. Afterwards, the incision was widened along the chest wall to the trachea. To flush the lungs, a 25-gauge needle was punctured into the trachea and a 19-gauge syringe containing 1.1 ml of phosphate-buffered saline (PBS) was inserted. The lungs were flushed with 1.1 ml of PBS and 800 µl of bronchoalveolar lavage fluid (BALF) were collected. BALF was centrifuged at 1164 x g for 5 min at 4°C and the supernatant was frozen at -80°C for subsequent analysis. The upper trachea was closed with a blunt clamp followed by perfusion of 20 ml of PBS through the heart using a 21-gauge blunt-tipped syringe to ensure systemic perfusion of the mouse. The left lung was ligated and removed, rapidly frozen in liquid nitrogen, and stored at -80°C. Furthermore, 10 ml of 4% buffered Zn-Formalin was perfused using a 21-gauge syringe for the right lung lobe, which was then removed and fixed overnight for subsequent (immuno)histological analyses.

### 2.5 Examination of lung damage

The samples were fixed in 4% buffered Zn-Formalin overnight, then, embedded in paraffin and sliced into 3 µm sections, which were stained with hematoxylin-eosin (HE). Specifically, the lung sections were deparaffinized, rehydrated, and stained with hemalum solution according to Mayer’s method (Carl Roth, Karlsruhe, Germany) for 10 minutes at room temperature (RT). After decolorization in rinse water for 10 minutes, the sections were counterstained with eosin (Carl Roth, Karlsruhe, Germany) for 3 minutes at RT. Histological damage in the HE-stained sections was assessed for each group in a blinded manner. To quantify the histopathological damage in the lungs, independent examiners assessed the lungs using the method described previously. Briefly, sections of lungs were examined for desquamation, dystelectasis/atelectasis, emphysema, congestion, interstitial thickness/infiltration with inflammatory cells, and bronchial exudate (33, 34).

### 2.6 Quantification of protein expression levels in BALF

To assess the severity of lung injury and the status of the lung barrier, levels of pro-inflammatory mediators in the BALF were evaluated. The concentrations of cleaved IL-1β and IL-6 in BALF were determined by performing mouse-specific enzyme-linked immunosorbent assays (ELISAs) using kits from R&D Systems (Minneapolis, USA) following the manufacturer’s instructions. The measurements were obtained using an Infinite M200 microplate reader (Tecan, Männedorf, Switzerland).

### 2.7 Ribonucleic acid isolation, reverse transcription and semi-quantitative polymerase chain reaction

RNA extraction from the lung homogenate that was obtained by mechanical disruption using the Precellys 24 Homogenizer (Bertin Technologies, Montigny-le-Bretonneux, France) and the buffer from the RNeasy assay (Qiagen, Hilden, Germany) was performed following the manufacturer’s protocol. To remove any remaining DNA, the sample was treated with the RNase-free DNase kit (Qiagen, Hilden, Germany). Qualitative and quantitative analysis of RNA was conducted with the Tecan’s NanoQuant Plate on the Spark M10 Microplate Reader (Tecan, Männedorf, Switzerland). For cDNA synthesis, the iScript™ cDNA Synthesis Kit (BioRad, Hercules, USA) was used according to the manufacturer’s instructions. Quantification of gene expression levels for *Muc5ac* (qMnuCED0061472), *Nlrp3* (qMmuCID0010647), and *Il-1β* (qMnuCED0045755) was performed using the PrimePCR SYBR Green Assay (BioRad, Hercules, USA) with specific primer sets for mouse. The housekeeping gene (control), *Gapdh* (qMnuCED0027467), was also quantified. *Gapdh* was chosen as control since no observed differences between aged, traumatized or sham groups were found. For all primers, the amplification specificities were confirmed by melting curve analysis, and no template controls were applied for detecting contamination or non-specific amplification. The PCR reaction was carried out with a total reaction volume of 25 µl and SYBR green qPCR Master Mix (BioRad) according to the manufacturer’s instructions. The reaction was performed using the C1000 Touch Thermal Cycler with the CFX96 Touch Real-Time PCR Detection System (BioRad, Hercules, USA). Finally, the relative expression level of each target gene was determined using the comparative threshold-cycle (CT) method (2^-DDCT^ method), which involved normalizing the expression of each target gene to that of *Gapdh*.

### 2.8 Western blotting

Lung tissue was homogenized in lysis buffer (Invitrogen™, FNN0021) at 4°C and centrifuged at 20.000 x g for 30 min at 4°C. The resulting supernatants were stored at -80°C for later analysis. Electrophoresis was performed on 20 µg protein lysate separated by a 12% polyacrylamide SDS gel and then transferred to a nitrocellulose membrane (Amersham-Buchler, Braunschweig, Germany). A rabbit polyclonal anti-NFkB p105/p50 (phospho S337) antibody (ab28849, 1:1000 dilution) was used to detect phosphorylated p50, and a rabbit monoclonal recombinant anti-NFkB p105/p50 antibody [E381] (ab32360, 1:2000 dilution) (Abcam, Cambridge, UK) was used to detect non-phosphorylated p50. Monoclonal beta-Actin antibody (sc-47778, 1:1000 dilution, Santa Cruz) was used as loading control for measuring beta-actin. Blots were blocked in a blocking buffer (10% non-fat dry milk in 1 mM Tris, 150 mM NaCl, pH 7.4) for one hour at RT, then incubated with primary antibodies in bovine serum albumin (BSA) containing 0.5% Tween 20 and 0.5% BSA at 4°C on a 35 rpm shaker overnight according to the manufacturer’s instructions. As secondary antibody a horseradish peroxidase-conjugated anti-rabbit IgG antibody (ab288151, 1:10000 dilution, Abcam) was subsequently applied for one hour at room temperature on the shaker. Proteins were detected using ECL™ Western blot detection reagent (GE Healthcare, Munich, Germany). After measuring the phosphorylated p50, nitrocellulose membrane was washed in TBS, then pp50 antibody was eluted for 2 × 15 minutes in stripping buffer (0.2 M glycine, 0.1% SDS, 1% Tween20, pH 2.2), and washed 3 × 5 minutes in TBST (0.05% Tween20 in TBS) on rocker with 50 rpm. The membrane was blocked for 1.5 hours in blocking buffer, and then incubated with p50 or β-actin antibodies overnight at 4°C. After incubation, the membrane was washed 2 × 15 minutes with TBST and 1 × 15 minutes with TBS (20 mM Tris-Base, 0.15 M NaCl, pH 7.6), and afterwards incubated with the secondary antibody as described above. The signals were digitized, and the ImageJ software was used to determine the integrated density of the individual bands for protein expression normalization to β-actin by densitometry using ImageJ software.

### 2.9 Immunohistology staining of NLRP3, HMGB1, ASC, Gasdermin D, SPC, active caspase-3 and neutrophil elastase

The lung tissue sections (3 μm) were subjected to dewaxing using Xylene (Merck, Darmstadt, Germany) or Roti Histol (Carl Roth, Karlsruhe, Germany) twice for 5 minutes and then rehydrated using descending alcohol series with 100%, 90%, and 70% concentrations sequentially for 2 x 5 minutes, then rinsed in distilled water for 2 x 5 minutes. Heat-induced epitope retrieval (HIER) was conducted using R-Universal epitope recovery buffer (Aptum, Kassel, Germany) in the 2100-Retriever (Prestige Medical, Blackburn, England) at 121°C for 20 minutes following the manufacturer`s manual. The slides were blocked and permeabilized with 150 µl of blocking solution (5% goat serum (Jackson immunoresearch), 0.05% TritonX, 0.05% Tween 20 in 1 x PBS) for 20 minutes at room temperature in a humidified incubation chamber. Afterwards, slides were washed in distilled water twice for 3 minutes. Lung sections were incubated with primary antibodies against Nucleotide-binding oligomerization domain (NOD)-like receptor (NLR) protein (NLRP)3 (1:200, rabbit anti-mouse, Cell signaling Technology, USA, #151015), Apoptosis-associated speck-like protein containing a CARD (ASC) (1:200, rabbit anti-mouse, Cell signaling Technology, USA, #67824), Gasdermin D (GSDMD) (1:200, rabbit anti-mouse, Cell signaling Technology, #39754), Prosurfactant protein C (SPC) (1:500, rabbit anti-mouse, Abcam USA, Ab90716), and High-mobility group box 1 protein (HMGB1) (1:200, rabbit anti-mouse, Abcam USA, Ab18256), which were diluted as suggested by the manufacturers in the Antibody Dilution Buffer (Dako Cytomation) and incubated overnight at 4°C in a humidified incubation chamber. Active caspase-3 (1:300, rabbit anti-mouse, anti-cleaved caspase-3 (Asp175), #9661, Cell Signaling Technology, USA), and neutrophil elastase (NE) (1:200, rabbit anti-mouse, Bioss, bs- 6982R, USA) antibodies were incubated for one hour at room temperature in a humidified incubation chamber. Incubation of primary antibodies was followed by washing according to the washing steps after blocking. Endogenous peroxidase was blocked by adding 3% hydrogen peroxide and incubation for 15 minutes at room temperature followed. After another washing step, the secondary antibody conjugated with horseradish peroxidase (Histofine Simple Stain Mouse MAX PO (R), Nichirei Biosciences Inc.) was incubated for 60 minutes at room temperature in a humidified incubation chamber according to manufacturers' instructions. This was followed by another washing procedure and 3-amino-9-ethylcarbazol (AEC, DCS Innovative Diagnostik-Systeme, Hamburg) was used to detect specific binding. Hematoxylin (Carl Roth, Karlsruhe, Germany), was used for counterstaining, and stained slides were mounted with mounting medium (Merck, 108562). Unstained sections for tissue background control were used. No primary antibody controls incubated with just the antibody diluent, without the primary antibodies were applied to determine if the secondary antibody is binding non-specifically to cellular components that do not contain the protein of interest, resulting in false positives or non-specific binding. Isotype control antibodies Normal Rabbit IgG (rabbit, Cell signaling Technology, USA, #2729) and Rabbit IgG, polyclonal - Isotype Control (rabbit anti-mouse, Abcam USA, Ab171870) were used in the control samples and incubated with the same concentrations under the same experimental conditions as applied for the corresponding primary antibody. Imaging was conducted using the Zeiss Axio Observer Z1 microscope with a 40 x objective except for ASC, that was captured with a 63 x objective (Zeiss, Göttingen, Germany). ImageJ software was used for evaluation, with counting of positively stained cells in 25 high-power fields (HPF).

### 2.10 Statistical analysis

The statistical analysis was conducted using GraphPad Prism 6 (GraphPad Software, Inc., San Diego, CA). Data were tested for normal distribution using Shapiro-Wilk normality test. Based on the histogram and Shapiro–Wilk test, the non-parametric Kruskal-Wallis test, which does not assume a normal distribution of the residuals, followed by Dunn’s *post hoc* test for the correction of multiple comparisons was applied. The results were presented as mean and standard error of the mean, and statistically significant differences are indicated as p-value less than 0.05.

## 3 Results

### 3.1 Impact of aging on lung damage after THFx

**Figure 1:**
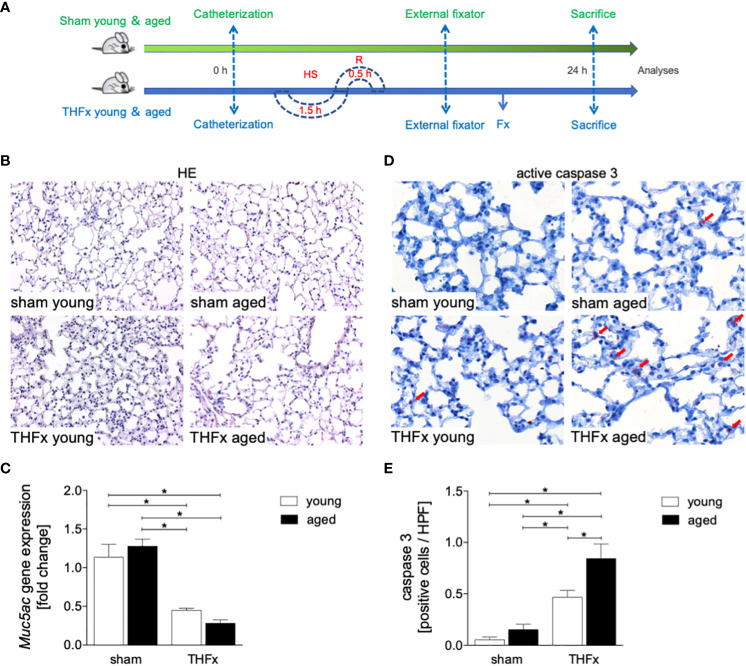
The effects of aging on lung damage after hemorrhagic shock (HS) and femoral fracture (Fx) were investigated. **(A)** The experimental design involved young (17 - 26 weeks) and aged (64 - 72 weeks) male C57BL/6J mice, as well as sham (green line) and trauma (blue line) groups. Trauma groups underwent pressure-controlled HS followed by resuscitation (R) with Ringer’s solution and Fx (via osteotomy) (THFx), while sham groups received catheterization and an external fixator but no THFx induction. Lung tissue samples were obtained 24 hours after the experiment, and Muc5ac gene expression and positive active caspase-3 cells were assessed. **(B)** Representative lung sections upon hematoxylin/eosin (HE) staining. **(C)** Relative gene expression of Muc5ac. **(D)** Representative lung sections upon the staining of activated caspase-3 with red arrows indicating caspase-3 positively stained cells, and **(E)** quantification of caspase-3 positively stained cells per high power field (HPF). n = 6 per group, * p < 0.05 between indicated groups.

**Figure 2:**
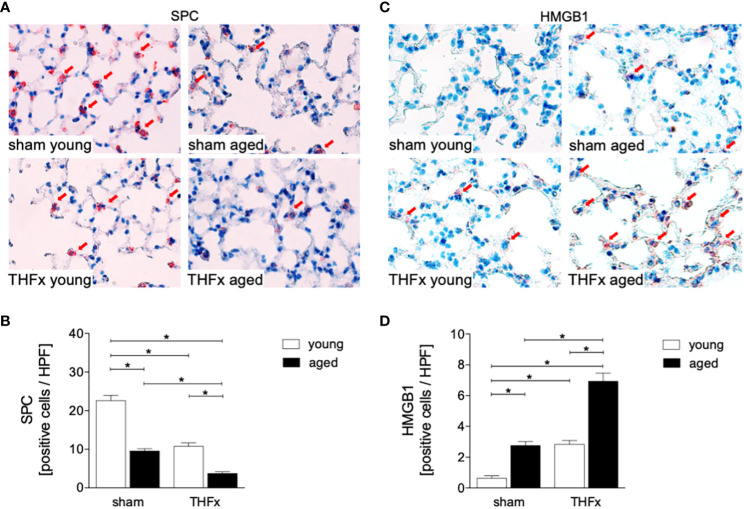
The impact of aging on Prosurfactant protein C (SPC) and High-mobility group box 1 protein (HMGB1) expression after hemorrhagic shock (HS) and femoral fracture (Fx). The experimental design included young (17 - 26 weeks) and aged (64 - 72 weeks) male C57BL/6J mice in both sham and trauma groups. The trauma groups underwent pressure-controlled HS followed by resuscitation (R) with Ringer’s solution and Fx (via osteotomy) (THFx), while the sham groups received catheterization and an external fixator but no THFx induction. After 24 hours, the mice were euthanized, and **(A)** SPC (red arrows) in lung tissue sections were quantified **(B)**. **(C)** Representative immunohistological HMGB1 staining (red arrows), and **(D)** positively stained cells per high power field (HPF). n = 6 per group, * p < 0.05 between indicated groups.

In this experiment, animals received an external fixator and underwent osteotomy and hemorrhagic shock with subsequent resuscitation as shown in **Figure 1A**. The study analyzed the differences in lung histomorphology, gene expression of the lung protective protein Muc5ac in homogenized lung tissue, prosurfactant protein C (SPC) expression as a marker of alveolar damage, and HMGB1 expression in lungs between groups (**Figures 1**, **2**). The results showed that THFx induced a strong histopathological lung injury with alveolar wall thickening and resulting alveolar space loss, with the most prominent damage observed in the THFx aged group compared to all other groups (**Figure 1B**). The histomorphological differences between the groups show increased lung injury in the THFx young group and further enhanced in the THFx aged group compared to both sham groups (lung injury score: sham young = 2, sham aged = 2, THFx young = 3, and THFx aged = 4). In addition, the gene expression of *Muc5ac* was significantly decreased in the THFx groups compared to the sham groups (p < 0.05, **Figure 1C**). In order to investigate apoptosis in aged lungs after trauma, active caspase-3 staining was used as a direct indicator of apoptosis (p < 0.05, **Figures 1D, E**). The results showed a significantly higher number of active caspase-3 positive cells (red arrows) in the THFx groups compared to the sham groups (p < 0.05, **Figure 1E**). Additionally, the number of caspase-3 positive cells was higher in the THFx aged group compared to the THFx young group (p < 0.05, **Figure 1E**). Prosurfactant protein C was significantly decreased in both aged groups compared to the corresponding young group (p < 0.05, **Figures 2A, B**). In addition, SPC presence was significantly decreased in the THFx groups compared to the sham groups (p < 0.05, **Figure 2B**). HMGB1 staining showed a significantly higher number of HMGB1 positive cells (red arrows) in the THFx groups compared to the sham groups (p < 0.05, **Figures 2C, D**). Additionally, the number of HMGB1 positive cells was higher in aged groups compared to the young groups (p < 0.05, **Figure 2D**).

### 3.2 Impact of aging on IL-6 and neutrophil infiltration after THFx

**Figure 3:**
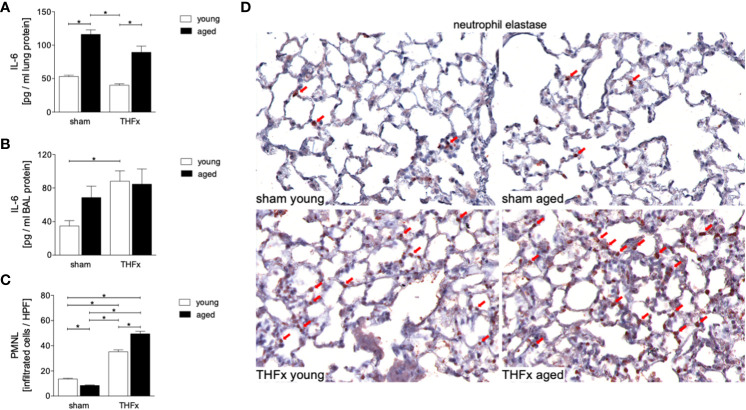
The impact of aging on interleukin (IL)-6 protein expression and neutrophil infiltration after hemorrhagic shock (HS) and femoral fracture (Fx) was investigated. The experimental design included young (17 - 26 weeks) and aged (64 - 72 weeks) male C57BL/6J mice in both sham and trauma groups. The trauma groups underwent pressure-controlled HS followed by resuscitation (R) with Ringer’s solution and Fx (via osteotomy) (THFx), while the sham groups received catheterization and an external fixator but no THFx induction. After 24 hours, the mice were euthanized, and IL-6 protein expression **(A)** in lung tissue homogenates and **(B)** in bronchoalveolar lavage (BAL) fluid was analyzed. **(C)** Quantification of Neutrophil elastase (NE) positively stained cells per high power field (HPF). **(D)** Representative immunohistological staining of NE as a marker of polymorphonuclear leukocytes (PMNL) in lung sections with red arrows indicating NE-positively stained cells. n = 6 per group, * p < 0.05 between indicated groups.

The study evaluated the protein levels of IL-6 in homogenized lung tissue and IL-6 protein concentration in the BALF. The results showed that protein expression of IL-6 in lung tissue was significantly increased in both aged groups, sham and THFx, compared to the corresponding young group (p < 0.05, **Figure 3A**). IL-6 protein expression in the BALF was significantly increased in THFx compared to the young sham group (p < 0.05, **Figure 3B**).

Neutrophil infiltration in the lung was quantified by immunohistological staining, showing a significantly increased infiltration of neutrophils in both THFx groups compared to the corresponding sham group (p < 0.05, **Figures 3C, D**). Additionally, the THFx aged group had significantly increased neutrophil infiltration compared to the THFx young group, indicating that aging can worsen the inflammatory response in the lung after trauma (p < 0.05, **Figures 3C, D**).

### 3.3 Impact of aging on NF-κB activation after THFx

**Figure 4:**
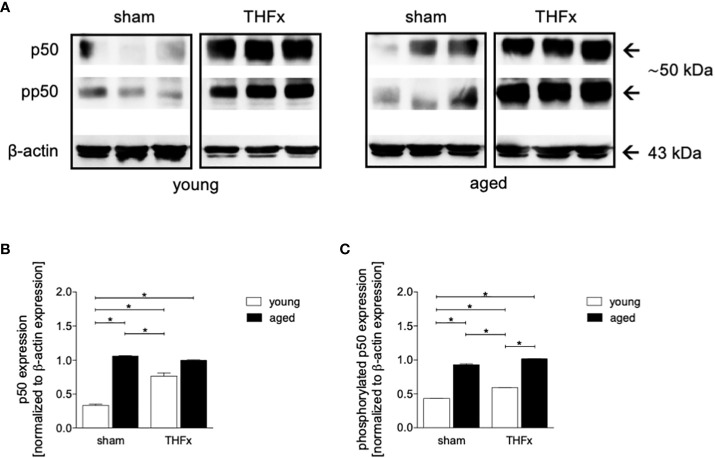
The effect of aging on NF-κB following hemorrhagic shock (HS) and femoral fracture (Fx) in young (17 - 26 weeks) and aged (64 - 72 weeks) male C57BL/6J mice is shown. The experimental design included sham and trauma groups, with the trauma groups subjected to pressure-controlled HS followed by resuscitation (R) with Ringer’s solution and Fx (via osteotomy) (THFx). The sham groups underwent catheterization and received an external fixator, but no THFx was induced. After 24 hours, the mice were euthanized, and samples were collected. **(A)** Representative western blot analysis of phosphorylated and non-phosphorylated p50 and β-actin, and quantification of **(B)** non-phosphorylated p50 and **(C)** phosphorylated p50 in young and aged sham and THFx mice. n = 4 per group, * p < 0.05 between indicated groups.

In this study, the impact of aging on the NF-κB signaling was investigated after THFx. Lung tissue homogenates were collected 24 hours after resuscitation, and western blot analysis was performed to measure phosphorylated and non-phosphorylated p50, as indicator for NF-κB activation. The results showed that non-phosphorylated p50 expression levels were significantly higher in both THFx groups compared to the young sham group (p < 0.05, **Figures 4A, B**). Additionally, the THFx young group showed significantly increased levels of non-phosphorylated p50 versus the young sham group, while there was no difference among the aged groups (p < 0.05, **Figures 4A, B**).

Phosphorylated p50 expression levels were significantly higher in both aged groups compared to their corresponding young groups (p < 0.05, **Figures 4A, C**).

### 3.4 Impact of aging on the expression of inflammasome components after THFx

**Figure 5:**
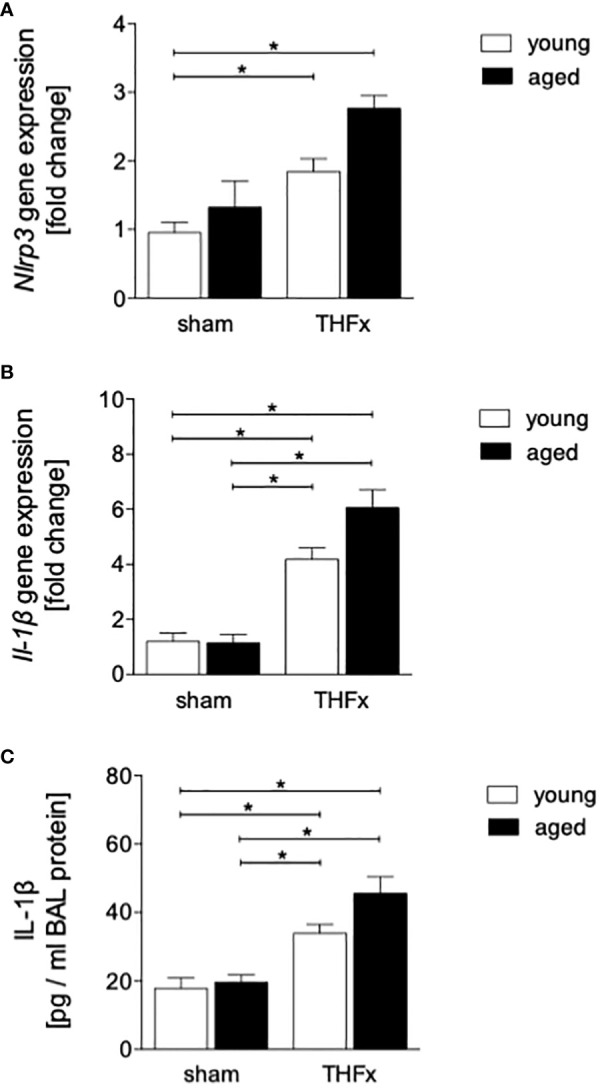
The effect of aging on the presence of inflammasome components after hemorrhagic shock (HS) and femoral fracture (Fx) in male mice. The experimental design included young (17 - 26 weeks) and aged (64 - 72 weeks) C57BL/6J mice in both sham and trauma groups. The trauma groups underwent pressure-controlled HS followed by resuscitation (R) with Ringer’s solution and Fx (via osteotomy) (THFx), while the sham groups underwent catheterization and received an external fixator, but no THFx was induced. After 24 hours, the mice were euthanized, and sampling was performed. **(A)** The relative gene expression of nucleotide-binding domain (NOD)-like receptor protein (NLRP)3, and **(B)** of interleukin (IL)-1β in lung tissue homogenates. **(C)** Quantification of IL-1β protein concentration in bronchoalveolar lavage (BAL) fluid. n = 6 per group, * p < 0.05 between indicated groups.

The gene expression of *Nlrp3* and *Il-1β* was assessed in homogenized lung tissue. Results showed that the expression of *Nlrp3* was significantly higher in THFx groups versus young sham group (p < 0.05, **Figure 5A**).

In addition, the relative gene expression of *Il-1β* in lung tissue was significantly higher in both THFx groups compared to their corresponding sham group (p < 0.05, **Figures 5B**).

The concentration of IL-1β in the BALF was significantly higher in THFx groups compared to their corresponding sham group (p < 0.05, **Figure 5C**).

**Figure 6:**
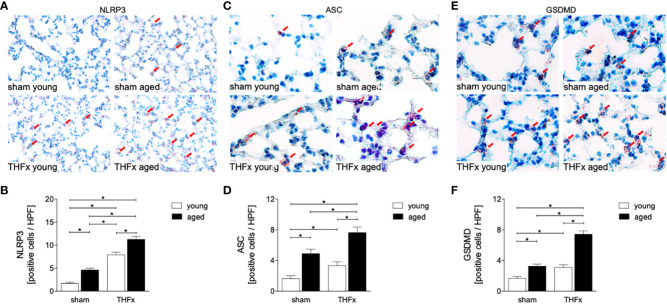
The impact of aging on Nucleotide-binding oligomerization domain (NOD)-like receptor (NLR) protein (NLRP)3, Apoptosis-associated speck-like protein containing a CARD (ASC), and Gasdermin D (GSDMD) expression after hemorrhagic shock (HS) and femoral fracture (Fx). The experimental design included young (17 - 26 weeks) and aged (64 - 72 weeks) male C57BL/6J mice in both sham and trauma groups. The trauma groups underwent pressure-controlled HS followed by resuscitation (R) with Ringer’s solution and Fx (via osteotomy) (THFx), while the sham groups received catheterization and an external fixator but no THFx induction. After 24 hours, the mice were euthanized, and **(A)** NLRP3 protein expression was assessed and quantified **(B)** in lung tissue sections. **(C)** Representative immunohistological staining of ASC and **(D)** quantification of ASC positively stained cells per high power field (HPF). **(E)** Representative immunohistological staining of GSDMD and **(F)** quantification of GSDMD positively stained cells per HPF. n = 6 per group, * p < 0.05 between indicated groups.

In order to further investigate the expression of inflammasome components in aged lungs after trauma, several markers including NLRP3, ASC and GSDMD were used (**Figure 6**). The results showed a significantly higher number of NLRP3 positive cells (red arrows) in the THFx groups compared to the corresponding sham group (p < 0.05, **Figures 6A, B**). The number of NLRP3 positive cells was significantly higher in the aged groups compared to the young groups, while the NLRP3 increase was significantly enhanced in the THFx aged group compared to all other groups (p < 0.05, **Figure 6B**).

The number of ASC positive cells (red arrows) was significantly higher in the THFx groups compared to the corresponding sham groups (p < 0.05, **Figures 6C, D**). The number of ASC positive cells was higher in the sham aged group compared to the sham young group (p < 0.05, **Figure 6D**). In the THFx aged group ASC was significantly higher compared to all other groups (p < 0.05, **Figure 6D**).

The number of GSDMD positive cells (red arrows) was significantly higher in the THFx groups compared to the corresponding sham groups (p < 0.05, **Figures 6E, F**). Furthermore, the number of GSDMD positive cells was higher in the sham aged group compared to the sham young group (p < 0.05, **Figure 6F**), and in the THFx aged group GSDMD was significantly higher compared to all other groups (p < 0.05, **Figure 6F**).

## 4 Discussion

The objective of our study was to explore the mechanisms that cause lung injury in young and aged mice after THFx. Trauma is the leading cause of death and disability worldwide (35). In young mice, femoral fracture following pressure-controlled hemorrhagic shock can cause increased uncontrolled inflammation in the lungs as well as disruption of the pulmonary barrier (33), yet the current research has not fully explained the effects of aging on lung injury and the pulmonary inflammatory response after THFx. Our study aimed to investigate how aging influences the typical NF-κB signaling pathway as well as the expression of inflammasome components that mediate the lung inflammatory response after THFx. Specifically, the study compared the response of young mice aged 17 - 26 weeks and old mice aged 64 - 72 weeks, to gain a better understanding of underlying mechanisms for age-related changes impacting lungs, especially in the context of trauma and hemorrhagic shock. In a recent study applying this model, it was shown that aged mice had significantly lower total bone and callus volume, decreased share of callus per total bone volume, less trabecular structures as well as the reduction of the elastic limit (31), thus, our model of femoral fracture mimics the human situation. Furthermore, it was demonstrated that THFx induced an uncontrolled inflammatory response and lung barrier breakdown in young mice (33), findings that support our hypothesis. This study’s results have the potential to guide future research and pre-clinical approaches for addressing these conditions in both younger and older populations.

The results suggest that trauma-induced inflammation and lung damage are exacerbated by the aging process through activation of the NF-κB signaling pathway and eventually inflammasome. The reduction in surfactant protein C in both THFx groups suggests an impaired lung protective response following trauma, which was further exacerbated in the THFx aged group (**Figures 1**, **2**). Reduced *Muc5ac* gene expression in the THFx groups supports increased lung damage following trauma (**Figure 1C**). Additionally, the increase in activated caspase-3 positive cells in both THFx groups indicates an increased apoptosis after THFx, which was more prominent in the THFx aged group compared to the THFx young group (**Figures 1D, E**). Similarly, the higher levels of HMGB1 and IL-6 suggest an amplified pro-inflammatory response in aging (**Figures 2**, **3**). The PMNL increase in both young and aged THFx groups compared to sham groups suggests that trauma-induced inflammation attracts more inflammatory cells to the lung, again, with the THFx aged group experiencing a more pronounced effect (**Figure 3C**). Enhanced expression of p50 and phosphorylated p50 in all aged groups indicates that the NF-κB signaling pathway is activated in aging (**Figure 4**). Also, the increased expression of inflammasome components as shown by increased IL-1β gene expression and concentration, as well as enhanced NLRP3, ASC and GSDMD expression in lungs following trauma suggests an involvement of the inflammasomes in the exaggerated inflammatory response in the lung following trauma in aged mice (**Figures 5**, **6**). Overall, the study highlights the role of aging in exacerbating the inflammatory response to trauma in lungs.

Trauma-induced cellular destruction with DAMPs release, as also demonstrated in the present study by HMGB1 increase, or translocation of pathogen-associated molecular patterns into the bloodstream is accompanied with the production and release of various inflammatory signals such as IL-1β, TNF-α and IL-6, as well as a significant increase in PMNL activation and infiltration in the lungs (36, 37). In line with these reports, our results demonstrate an increase in the production as well as in the release of pro-inflammatory cytokines, including IL-6 and IL-1β after THFx. Typically, the release of inflammatory mediators after trauma is linked to an enhanced PMNL activity and infiltration in lungs. Störmann et al. found a significant increase in PMNL infiltration in the lungs after polytrauma in pigs (36), which was attributed to the lipid mediator leukotriene (LT)B4 that activates and attracts PMNL to the lungs to participate in post-traumatic lung inflammation (36, 38). Kalsotra et al. have shown that both LTB4 levels and PMNL infiltration were increased in the lungs of mice with brain contusion, and furthermore, were attributed to pulmonary complications following brain injury (39). It was also demonstrated before that IL-6 plays a significant role in inflammatory conditions (7, 12, 40, 41). In our previous *in vivo* study, we found significantly increased IL-6 levels in lung and liver samples after polytrauma compared to sham (42). In line with those and other reports, here, a significant PMNL infiltration into the lungs and an elevated concentration of IL-6 in BAL after THFx was observed (**Figure 3**). Thus, the development of lung injury and apoptosis in lung cells following THFx is closely associated with the release of inflammatory mediators. Studies using mouse models of indirect acute lung injury (ALI) induced by hemorrhagic shock and sepsis showed that lung inflammation is characterized by the caspase-3-dependent apoptosis of pulmonary epithelial cells (43, 44). The project presented here revealed an increase in caspase-3 levels and SPC indicating lung damage in mice with THFx (**Figures 1**, **2**), which is in consistence with the findings of Zhang et al. (45).

The transcription factor NF-κB complex consists of a group of isoforms that regulate genes involved in the inflammatory response, as well as other biological processes such as cell growth and survival (46). Activation of NF-κB is closely linked to the development of various diseases, including cancer, autoimmune disorders, and chronic inflammation (47). Traumatic injury as also observed in this study, infections, and aging can lead to the activation of the NF-κB pathway and promotion of inflammatory signals (48). Apart from the NF-κB pathway induced by trauma, various DAMPs triggered by trauma, such as mitochondrial DNA, HMGB1, and ATP, can activate Nod-like receptors (NLRs) and promote the formation and activation of NLRP3 (49). The activated NLRP3 inflammasome leads to caspase-1 activation, resulting in the release of mature forms of IL-1β and IL-18 that promote exaggerated inflammatory responses in the circulation and peripheral tissues, leading to cell death, a process known as pyroptosis (50–53). Animal studies have also demonstrated that NLRP3 inflammasomes in lung endothelial cells and alveolar macrophages (AMs) are increasingly activated after hemorrhagic shock, leading to lung inflammation and increased IL-1β expression (54–56). Additionally, the activation of endothelial cell pyroptosis as shown by Yang et al. resulted in increased inflammation and lung damage in mice undergoing HS and LPS challenge (55). Furthermore, Xu et al. reported an increase in NLRP expression in lung endothelial cells after HS, leading to higher IL-1β levels detected in BAL (56). In consistence with these studies, we demonstrated here a significant increase in lung inflammation after THFx, as indicated by a significantly upregulated gene expression of *Nrlp3* in the lung and an increased concentration of IL-1β in BAL (**Figure 5**). Also, enhanced protein expression of NLRP3, ASC and GSDMD after trauma (**Figure 6**), and further amplification of these trauma-induced inflammasome-related mediators support the hypothesized of a possible activation of inflammasomes after trauma, and indicate that the heightened expression of inflammasome components may be linked to lung damage after THFx.

As all organisms age, there is a gradual and ongoing increase in chronic inflammation that is not caused by infection or injury (57). Senescent tissues produce various molecules, collectively called senescence-associated secretory phenotype (SASP) such as pro-inflammatory cytokines among others IL-1 (58). These molecules promote the further release of inflammatory cytokines and affect the local environment, leading to systemic and local inflammation through the blood circulation (23). Neutrophil elastase a marker for neutrophils is also associated with aging. In the brain tissue of Alzheimer’s disease patients, the activity and concentration of neutrophil elastase gradually increase with age (59). However, the ability of neutrophils to phagocytosis and chemotaxis decreases with aging (60). Aged mice have been shown to have a high frequency of transendothelial migration of neutrophils, which can infiltrate the lungs and cause remote lung injury (RLI) (61). Corberand and colleagues found that there is an increased proportion of neutrophils in bronchoalveolar lavage fluid of healthy older adults (62). Additionally, the pro-inflammatory pulmonary mononuclear macrophage subpopulation increases with age, leading to the secretion of pro-inflammatory cytokines including IL-6, which contribute to inflammation (63, 64). This is in line with our findings showing enhanced pro-inflammatory cytokine levels in lungs from aged mice. According to Balistreri et al., the inflammatory response to trauma in aging mice may be exacerbated due to a stronger activation of NF-κB, indicating that aging may worsen the response through this signaling pathway (65). Similarly, Zhao et al. suggested that NF-κB activity is upregulated in aging mice (66). Also, data from our study indicate that aged mice have stronger NF-κB activation compared to young mice (**Figure 4**). Notably, the increased levels of apoptotic markers in aging mice are related to the accumulation of mutant mRNA in senescent cells, and the results of Kujoth et al. suggest that aged mice expressed higher levels of caspase-3 in multiple organs (67). In line with these reports, our results show a significant increase in caspase-3 levels in aged mice after trauma (**Figures 1D, E**). Also, the NLRP3 inflammasome is involved in the pathogenesis of many age-related diseases. Trauma-induced and aging-related DAMPs are known to induce NLRP3 and IL-1β cleavage (18, 68, 69). It is noteworthy that the discoveries shown in this study disclosed higher levels of gene expression for *Il-1β* and *Nlrp3* as well as enhanced NLRP3, ASC and GSDMD protein expression in traumatized elderly mice, alongside elevated IL-1β protein levels in BAL fluid (**Figures 5**, **6**). Furthermore, this experiment detected a more significant degree of neutrophil infiltration, and lung damage indicated by SPC and HMGB1 in aged lung tissues following THFx compared to younger mice. These findings imply that the aging process intensifies the production of inflammatory agents and neutrophil infiltration in the lung post THFx, resulting in a more severe inflammatory response and damage of the lung tissue.

This study has several limitations that should be acknowledged. Firstly, only male mice and not female mice were used in our experiments. Therefore, the conclusion can only be drawn in the male population, and we need to take factor sex into account in future studies. Secondly, in the clinical setting, geriatric patients may have many relatively complex chronic inflammatory diseases, and we have chosen healthy mice, which may have some impact on the results of studies on inflammation. Moreover, the model we used to induce femur fracture and subsequent immune responses in mice may not fully reflect the clinical situation in humans. The clinical scenario usually involves traumatic injury, hemorrhagic shock caused by uncontrolled bleeding, and femur fixation, whereas our model involves pressure-controlled hemorrhagic shock followed by the insertion of a fixator and femoral osteotomy. Moreover, we did not ventilate with intubation during the anesthesia, and the animals are allowed to perform fully weight-bearing activities directly after they awaken from anesthesia, which is very different from the clinical situation. Another limitation of this study is the lack of protein analyses via western blot. Additionally, we only analyzed lung tissue and BAL fluid from a single time point, which limits the generalizability of our findings to other time points. Future studies examining immune responses at multiple time points and prolonged observation of mice after trauma, are necessary to provide more reliable conclusions.

The study aimed to understand how aging affects lung injury and the pulmonary inflammatory response after THFx in mice. Results showed that aging exacerbates trauma-induced inflammation and lung damage through activation of the NF-κB signaling pathway and increased expression of inflammasome components. Thus, the findings highlight the importance of understanding the mechanisms that contribute to an immunosenescent state in aging and how this may impact the inflammatory response following trauma, and furthermore suggests that the vulnerability to dysregulated post-traumatic immune responses increases with age, which may contribute to a higher risk of trauma-induced organ damage, infectious complications, and increased morbidity and mortality in older patients.

## Data availability statement

The raw data supporting the conclusions of this article will be made available by the authors, without undue reservation.

## Ethics statement

All animal studies were carried out in strict accordance with the German Animal Law and with the permission of the local authorities in Lower Saxony, Germany (approval number: 33.12-42502-04-17/2491). The study was conducted in accordance with the local legislation and institutional requirements.

## Author contributions

YZ: Data curation, Formal analysis, Investigation, Methodology, Validation, Visualization, Writing – original draft. FM: Investigation, Methodology, Writing – review & editing. KK: Methodology, Writing – review & editing. JB: Writing – review & editing. AW: Writing – review & editing. CN: Conceptualization, Funding acquisition, Resources, Supervision, Writing – review & editing. KB: Investigation, Methodology, Supervision, Validation, Writing – original draft. BR: Conceptualization, Data curation, Formal analysis, Funding acquisition, Resources, Supervision, Validation, Visualization, Writing – original draft.
